# The 50% effective dose of remimazolam combined with different doses of esketamine for painless gastroscopy

**DOI:** 10.1038/s41598-025-97649-1

**Published:** 2025-04-14

**Authors:** Li Zhao, Xuelei Zhou, Linlin Chen, Wei Mao, Yiping Guo, Xianchun Liu, Longyi Zhang, Ying Xie, Linji Li

**Affiliations:** 1 Department of Anesthesiology, The Second Clinical Medical College, North Sichuan Medical College, Beijing Anzhen Nanchong Hospital of Capital Medical University & Nanchong Central Hospital, Nanchong, China; 2 Nanchong Center for Disease Control and Prevention, Nanchong, China

**Keywords:** Painless gastroscopy, Gastrointestinal endoscopy, Remimazolam, Esketamine, Median effective dose, Drug development, Gastroenterology

## Abstract

Objective: Remimazolam is a novel benzodiazepine sedative that provides effective sedation, stable haemodynamics, and minimal adverse effects during intravenous general anaesthesia. The aim of this study was to determine the 50% effective dose (ED50) of remimazolam combined with different doses of esketamine for painless gastroscopy and to evaluate the efficacy and safety of this combination. Methods: This was a randomised, double-blind, up-and-down sequential allocation study. Patients undergoing painless gastroscopy who met all the inclusion criteria and did not meet any of the exclusion criteria were randomised in a 1:1:1 ratio into the ES0 group (0 mg/kg of esketamine), ES1 group (0.2 mg/kg of esketamine), and ES2 group (0.4 mg/kg of esketamine). The initial dose of remimazolam was 0.3 mg/kg in each group, with the dose increased or decreased by 0.05 mg/kg for the subsequent patient based on the success or failure of sedation in the previous patient. The trial was concluded when seven successful failure crossovers were achieved. The ED50 and 95% confidence intervals (CI) of remimazolam were calculated using Probit regression. Haemodynamic parameters, time to induction of anaesthesia, time to gastroscopy, time to awakening from anaesthesia, and adverse events were recorded. Results: A total of 59 patients were included in the final analysis: 19 in the ES0 group, 23 in the ES1 group, and 17 in the ES2 group. The ED50 (95% CI) of remimazolam in the ES0, ES1, and ES2 groups was 0.344 (0.302–0.389) mg/kg, 0.289 (0.249–0.328) mg/kg, and 0.193 (0.145–0.239) mg/kg, respectively. Additionally, the ES1 and ES2 groups exhibited more stable haemodynamics compared to the ES0 group. However, the ES1 and ES2 groups had significantly longer recovery times than the ES0 group. The incidence of hypotension was higher in the ES0 group compared to the ES1 and ES2 groups. Conclusion: The ED50 of remimazolam combined with 0 mg/kg, 0.2 mg/kg, and 0.4 mg/kg of esketamine for induction of anaesthesia during painless gastroscopy was 0.344 mg/kg, 0.289 mg/kg, and 0.193 mg/kg, respectively. Combining esketamine with remimazolam for induction of anaesthesia during painless gastroscopy offers advantages in terms of haemodynamic stability and reduced adverse effects.

## Introduction

With the advancement of medical care, the demand for painless gastroscopy among patients has increased year by year^1–3^. Gastroscopy has significant diagnostic value for conditions such as gastritis, peptic ulcers, and gastro-oesophageal tumours^4^. However, traditional gastroscopy procedures often result in patient discomfort due to painful stimuli, which can lead to interruptions and, consequently, missed or inaccurate diagnoses. Numerous studies have shown that painless gastroscopy significantly shortens operation time, reduces stress responses, and minimises pain during the examination compared to conventional gastroscopy^5,6^.

Painless gastroscopy typically requires an appropriate depth of anaesthesia. Currently, commonly used drug regimens include propofol or etomidate in combination with opioids^7,8^. Although propofol is widely used, it is associated with adverse effects such as hypotension, bradycardia, respiratory depression, and injection pain^9,10^. Etomidate, while causing less respiratory and circulatory depression, carries risks such as muscle tremors, postoperative nausea and vomiting, and suppression of adrenocortical function^11,12^. Sufentanil, although effective for analgesia, increases the risks of hypotension, respiratory depression, and postoperative cognitive dysfunction^13,14^. Therefore, it is crucial to identify an anaesthetic drug combination that ensures safe and effective painless gastroscopy.

Remimazolam, a novel benzodiazepine sedative, has the advantages of a rapid onset of action, fast metabolism, minimal accumulation, and a low impact on cognitive brain function^15,16^. It is hydrolysed to inactive metabolites by plasma esterases and has minimal effects on hepatic and renal function. Clinical studies have demonstrated that remimazolam’s anaesthetic effect during painless gastroscopy is comparable to propofol, but with fewer respiratory and circulatory adverse effects^17,18^.

Esketamine, an isomer of ketamine, has stronger analgesic and sedative effects than ketamine, while causing fewer psychiatric and circulatory adverse effects^19,20^. Studies have shown that esketamine can provide effective analgesia while maintaining stable haemodynamics and causing fewer respiratory adverse events^21,22^.

Recent studies suggest that the combination of remimazolam and esketamine for intravenous general anaesthesia provides effective sedation and analgesia with fewer adverse events. However, there remains a paucity of data regarding the appropriate dosage for this combination^23,24^. Therefore, the present study was designed to address this gap. This study aimed to determine the 50% effective dose (ED50) of remimazolam combined with different doses of esketamine for painless gastroscopy and to evaluate its anaesthetic effects, providing guidance for clinical practice.

## Methods

### Ethical approval

This was a single-centre, randomised, double-blind, up-down sequential allocation trial. The study was approved by the Ethics Committee of Nanchong Central Hospital (2022, trial (003) No.) and registered in the China Clinical Trial Registry (Registration time: 21/02/2023; Available at https://www.chictr.org.cn/bin/project/edit?pid=178987; Registration Number: ChiCTR2300068488). Each patient provided informed consent before painless gastroscopy. We confirm that our study complies with the Declaration of Helsinki.

### Participants

A total of 84 adult patients who underwent painless gastroscopy between June 2023 and October 2023 were recruited for this study. Inclusion criteria were: age 18–65 years, body mass index (BMI) 19–28 kg/m^2^, and American Society of Anesthesiologists (ASA) class I–II. Exclusion criteria included: complex endoscopic operations such as gastric polypectomy, severe cardiopulmonary disease, abnormalities in liver and kidney function, a history of allergy to the study medications, and anticipated difficult airway.

### Randomisation and blinding

Eligible patients were randomly allocated to the ES0 group (0 mg/kg of esketamine), ES1 group (0.2 mg/kg of esketamine), and ES2 group (0.4 mg/kg of esketamine) in a 1:1:1 ratio. Allocation details were concealed in opaque sealed envelopes. A non-blinded nurse prepared the study medication in uniform looking 10 mL syringes according to the patient’s weight, labeled only with the patient’s number.The syringes in the ES0 group were filled with saline in the same volume as in the ES1 and ES2 groups. All patients, surgeons, and other investigators were blinded to the group assignment.

### Anaesthesia

All patients fasted for 8 h before the procedure. Upon entering the gastroscopy room, monitoring was initiated to continuously measure the electrocardiogram, pulse oximetry (SpO₂), non-invasive arterial blood pressure, and heart rate (HR). Peripheral venous access was secured, and Ringer’s lactate solution was infused. The patient was placed in the left lateral position, and oxygen was administered via a nasal cannula at a flow rate of 3 L/min. Anaesthesia induction began after the patient was oxygenated to an oxygen saturation of 100%. Intravenous esketamine and remimazolam were administered sequentially. Gastroscopy was initiated when the Modified Observer’s Alertness/Sedation scale (MOAA/S) was ≤ 1. During the procedure, MOAA/S was assessed every minute to ensure sustained sedation (score ≤ 1). If the score exceeded 1 at any time, additional remimazolam (2.5–5 mg) was administered. Vital signs continued to be monitored after the examination, and the time at which the patient’s MOAA/S score reached 5 was recorded as the point of awakening.

### Determination of ED50

The ED50 of remimazolam was determined using Dixon’s up-and-down sequential allocation method^25^. The initial dose of remimazolam in each group was 0.3 mg/kg. Sedation failure was defined as an MOAA/S score *>* 1 within 3 min of starting gastroscopy, after which the dose for the next patient was increased by 0.05 mg/kg. Otherwise, the dose was decreased by 0.05 mg/kg. The trial was concluded when there were seven successful and failed crossovers, at which point the sample size was considered sufficient^26^.

### Study outcomes

The primary outcome was the ED50 of remimazolam. Secondary outcomes included the following: patient baseline characteristics (age, gender, height, weight, BMI, ASA classification, etc.), anaesthetic induction time (Dosing was initiated until MOAA/S score ≤ 1), operative time, awakening time (End of gastroscopy to MOAA/S score = 5), endoscopist satisfaction, and patient satisfaction. Haemodynamic parameters were recorded at T1 (baseline), T2 (start of gastroscopy), T3 (after 2 min), T4 (at the end of the procedure), and T5 (at awakening). Adverse events occurring between the start of the examination and the patient’s awakening were also recorded. These included hypertension, hypotension, tachycardia, bradycardia, and hypoxaemia. Blood pressure was classified as hypertension or hypotension if it increased or decreased by more than 20% of the baseline value. Bradycardia or tachycardia was defined as an HR of < 50 or > 100 beats/min^27^. Hypoxaemia was defined as an SpO₂ level of less than 90% and was managed using mandibular support, increased oxygen flow, mask pressure oxygenation, or, if necessary, emergency intubation.

###  Statistical analysis

The sample size for this study was based on the sequential allocation method. According to this principle, ED50 can be accurately calculated when the sample size reaches seven crossings^28^. ED50 and ED95 were estimated using probabilistic regression analysis of SPSS. Normally distributed data were expressed as mean ± standard deviation. Between-group comparisons were performed using one-way ANOVA, while repeated measures ANOVA was used for within-group comparisons. Non-normally distributed data were expressed as median and interquartile range, analysed using the Kruskal-Wallis H test. Qualitative data were expressed as frequencies and percentages, analysed using the chi-square test. Ordinal data (e.g., satisfaction scores) were analyzed using the Kruskal-Wallis test. A *P*-value of *<* 0.05 was considered statistically significant.

## Results

**Fig. 1:**
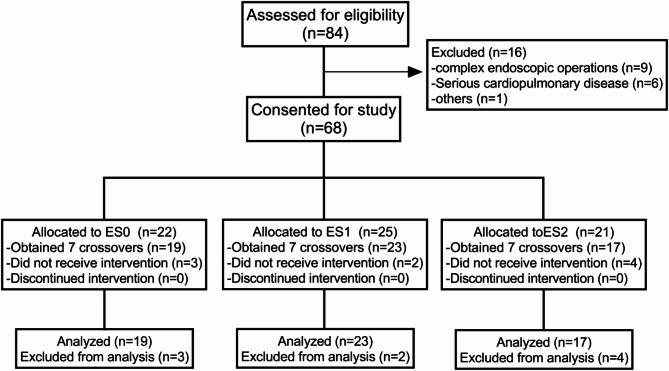
Study flow chart.

The flow chart of this study is shown in Fig. 1. We assessed 84 patients for eligibility between April and June 2023. Twenty-five patients were excluded, of whom 16 did not meet the eligibility criteria, and nine patients were withdrawn from the study. A total of 59 patients were included in the final analysis, with 19 in the ES0 group, 23 in the ES1 group, and 17 in the ES2 group, and seven crossovers occurring in each group.

The baseline characteristics for the three groups of patients are shown in Table 1. There were no significant differences in the baseline characteristics of gender, age, ASA classification, BMI, hypertension, and diabetes among the three groups (*P* > 0.05).

**Table 1:** Baseline characteristics of the enrolled patients.

Item	ES0(*n* = 19)	ES1(*n* = 23)	ES2(*n* = 17)	*P*
Sex (M/F))	7/12	10/13	8/9	*P* = 0.846
Age (y)	50.4 ± 10.6	50.6 ± 10.4	46.3 ± 13.3	*P* = 0.442
BMI(kg/m^2^)	23.2 ± 2.5	23.2 ± 2.4	23.5 ± 2.4	*P* = 0.923
ASA(I/II)	4/15	3/20	5/12	*P* = 0.614
Hypertension	2(10.5%)	1(4.4%)	1(5.9%)	*P* = 0.82
Diabetes	4(21.1%)	1(4.3%)	1(5.9%)	*P* = 0.26
Duration of procedure (seconds)	173.1 ± 144.3	148.6 ± 46.9	223.8 ± 128.1	*P* = 0.111

Data are presented as mean ± SD or number (percentage of patients).

BMI, body mass index; ASA, American Society of Anesthesiologists.

**Fig. 2:**
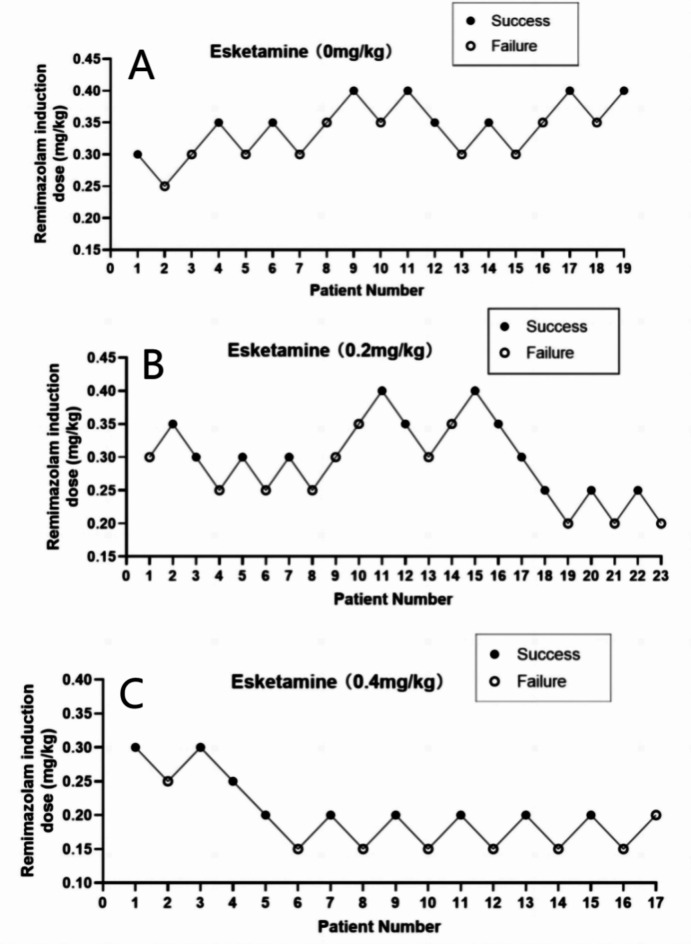
Patient up-down chart.

Figure 2 shows the ED50 determination of remimazolam in the three groups. The ED50 of remimazolam for painless gastroscopy was 0.344, 0.289, and 0.193 mg/kg in the ES0, ES1, and ES2 groups, respectively (Table 2). The ED50 of remimazolam was significantly lower in the ES2 group compared to the ES0 and ES1 groups (*P <* 0.05).

**Table 2:** ED50 and ED95 and their 95% CI.

	ES0 (*n* = 19)	ES1 (*n* = 23)	ES2 (*n* = 17)
ED50(mg/kg)	0.344 (0.302–0.389)	0.289 (0.249–0.328)	0.193 (0.145–0.239)* (*P* < 0.001) # (*P* = 0.003)
ED95(mg/kg)	0.442 (0.395–0.563)	0.387 (0.344-0.5)	0.29 (0.243–0.408)

Data are presented as mean (95%CI). #: Significant difference compared to ES1 group (*P* < 0.05); *: Significant difference compared to ES0 group (*P* < 0.05).

The hemodynamic data of the patients are shown in Tables 3 and 4. HR results for the three groups of patients are as follows. In the intragroup comparisons of the ES0, ES1, and ES2 groups, the HR at time points T2 and T3 were significantly increased compared to T1 (*P* < 0.05). In the intergroup comparisons, the HR of the ES1 and ES2 groups at time point T2 were significantly lower than that of the ES0 group (*P* = 0.016). There were no statistically significant differences in HR at the other time points (*P* > 0.05).

**Table 3:** HR data.

Item	Time	ES0 (*n* = 19)	ES1 (*n* = 23)	ES2 (*n* = 17)
HR (beats/min)	T1	76.68 ± 8.88	74.09 ± 9.25	69.88 ± 11.75
	T2	89.21 ± 9.05 # (*P* < 0.01)	81.65 ± 9.81 * (*P* = 0.016)# (*P* = 0.01)	82.06 ± 10.72 * (*P* = 0.034) # (*P* < 0.01)
	T3	85.94 ± 9.52 # (*P* < 0.01)	80.66 ± 8.40 # (*P* = 0.11)	81.97 ± 11.30 # (*P* < 0.01)
	T4	79.21 ± 10.65	76.04 ± 9.76	77.53 ± 12.54
	T5	78.26 ± 9.33	75.22 ± 9.99	74.35 ± 11.88

Data are presented as mean ± SD. #: Significant difference compared to T1 (*P* < 0.05); *: Significant difference compared to ES0 group (*P* < 0.05). T1 (at baseline), T2 (at the start of gastroscopy), T3 (after 2 min), T4 (at the end), and T5 (at awakening) were recorded. Data were tested using Repeated Measures ANOVA.

**Table 4:** MAP data.

Item	Time	ES0 (*n* = 19)	ES1 (*n* = 23)	ES2 (*n* = 17)
MAP (mmHg)	T1	88.89 ± 10.89	87.91 ± 10.94	81.69 ± 12.20
	T2	78.84 ± 13.93 # (*P* < 0.01)	88.64 ± 14.62 * (*P* = 0.027)	85.82 ± 12.87
	T3	74.39 ± 15.06 # (*P* < 0.01)	82.08 ± 10.45	82.98 ± 11.20
	T4	69.33 ± 11.07 # (*P* < 0.01)	77.43 ± 11.00 * (*P* = 0.02) # (*P* < 0.01)	80.41 ± 10.45 * (*P* = 0.03)
	T5	69.95 ± 11.30 # (*P* < 0.01)	76.67 ± 10.98 # (*P* < 0.01)	80.10 ± 11.04 * (*P* = 0.08)

Data are presented as mean ± SD. #: Significant difference compared to T1 (*P* < 0.05); *: Significant difference compared to ES0 group (*P* < 0.05). T1 (at baseline), T2 (at the start of gastroscopy), T3 (after 2 min), T4 (at the end), and T5 (at awakening) were recorded. Data were tested using Repeated Measures ANOVA.

The results of MAP during painless gastroscopy for the three patient groups are as follows. In the ES0 group, MAP at time points T2, T3, T4, and T5 was significantly lower compared to T1 (all *P* < 0.001). In the ES1 group, MAP at time points T4 and T5 was significantly lower compared to T1 (*P* < 0.001). In the ES2 group, there were no statistically significant differences in MAP across all time points (*P* > 0.05). In between-group comparisons, at time point T2, MAP in the ES0 group was significantly lower than that in the ES1 group (*P* = 0.027). At time point T4, MAP in the ES0 group was significantly lower than that in both the ES1 and ES2 groups (*P* = 0.02, *P*  = 0.003). At time point T5, MAP in the ES0 group was significantly lower than that in the ES2 group (*P* = 0.008).

The secondary outcome indicators of this study are shown in Table 5. In terms of time to awaken from anaesthesia, patients in the ES1 and ES2 groups had significantly longer awakening times compared to the ES0 group (*P* = 0.004). Regarding endoscopist satisfaction, there was no statistically significant difference between the ES0 and ES1 groups, but the ES2 group had significantly lower satisfaction compared to both the ES0 and ES1 groups. In terms of patient satisfaction, there was no statistically significant difference between the ES0 and ES1 groups, but the ES2 group had significantly lower satisfaction compared to the ES0 group. In the comparison of adverse events, there was a significant reduction in the incidence of hypotension in the ES1 and ES2 groups compared to the ES0 group, and no statistically significant differences in other adverse effects.

**Table 5:** The secondary outcomes in this study.

Item	ES0(*n* = 19)	ES1(*n* = 23)	ES2(*n* = 17)	*P*
Induction time (s)	90.1 ± 18.6	93.5 ± 12.2	94.0 ± 15.9	*P*=0.706
Recovery time (s)	460.8 ± 210.5	677.3 ± 211.4*	795.8 ± 431.1*	*P*=0.004
Endoscopist satisfaction (1–5)	5(5–5)	5(4–5)	4(4–5)*#	*P*=0.015
Patient satisfaction (1–5)	5(4–5)	5(4–5)	4(4–5)*	*P*=0.008
Adverse events				
Hypertension, n(%)	0(0%)	2(8.7%)	4(23.53%)	*P*=0.063
Hypotension, n(%)	14(73.7%)	6(26.1%)*	2(11.8%)*	*P <* 0.01
Tachycardia, n(%)	0(0%)	0(0%)	0(0%)	
Bradycardia, n(%)	4(21.1%)	1(4.3%)	4(23.5%)	*P*=0.157
Hypoxaemia, n(%)	1(5.3%)	1(4.3%)	1(5.9%)	*P*=1
Injection pain, n(%)	1(5.3%)	3(13.0%)	2(11.8%)	*P*=0.759

Data are presented as mean ± SD, median (25th to 75th percentiles), or number (percentage of patients). #: Significant difference compared to ES1 group (*P* < 0.05); *: Significant difference compared to ES0 group (*P* < 0.05). Satisfaction ratings range from a minimum of 1 to a maximum of 5.

## Discussion

The use of painless gastroscopy techniques is becoming more widespread as the need for comfort medicine in clinical practice increases. Moderate to deep sedation must be maintained during painless gastroscopy. Currently, a combined anaesthetic regimen of propofol with opioid analgesics is predominantly used in clinical practice. This combination usually achieves good sedation and analgesia; however, both propofol and opioids have strong inhibitory effects on respiration and circulation. This increases the likelihood of respiratory depression, hypoxaemia, and hypotension during the examination^29,30^. The anaesthetic regimen of remimazolam combined with esketamine, which has a rapid onset of action, good sedation and analgesia, as well as fewer inhibitory effects on respiration and circulation, is a potentially excellent alternative for painless gastroscopy^31^.

The sequential allocation method is effective for evaluating the ED50 of drugs in small sample cases, so it has been widely used in anaesthesia study design^32^. In the present study, by designing three different doses of esketamine, it was found that a higher dose of esketamine reduced the ED50 of remimazolam for anaesthesia induction during painless gastroscopy. In Hua et al.‘s study, a higher dose of esketamine in geriatric painless gastroscopy reduced the ED50 of propofol, which was similar to the results obtained in the present study^33^.

The haemodynamic results of this study demonstrate positive implications. HR increased significantly in all three groups of patients after the start of gastroscopy, but the increase in HR was significantly greater in the ES0 group than in the ES1 and ES2 groups. This indicates that gastroscopic manipulation stimuli lead to an increase in HR in patients, but esketamine’s analgesic effect mitigates this response, resulting in a relatively smaller increase and more stable HR. In certain time comparisons, both the ES1 and ES2 groups exhibited a more stable MAP than the ES0 group. The possible mechanism is that esketamine indirectly excites the sympathetic nervous system, reducing the fall in blood pressure^34^. Therefore, we recommend the use of remimazolam combined with esketamine for more stable haemodynamics during painless gastroscopy. However, because of the higher incidence of hypertension in the ES2 group, we wish to emphasize the unique advantages of the ES1 group (0.2 mg/kg esketamine), which balances hemodynamic stability with clinical utility.

Our study found that patients in the ES1 and ES2 groups had a longer time to awaken. Previous studies have reported that the half-life of remimazolam is 0.6–0.9 h, which is comparable to propofol^35^. In contrast, esketamine has a half-life of 3–5 h, which may have contributed to the significant prolongation of awakening time in patients in the ES1 and ES2 groups. In addition, esketamine is slowly metabolised in vivo, and after conversion to desmethyl ketamine by hepatic enzymes, it retains a potency equivalent to 1/5 to 1/3 that of esketamine, along with a longer half-life (8–9 h). Given the dose-dependent prolongation of awakening time with esketamine, we recommend using doses below 0.2 mg/kg for short procedures like gastroscopy. This approach balances the benefits of hemodynamic stability with the need for rapid recovery, though further studies are needed to confirm optimal dosing.

Overall, remimazolam in combination with esketamine demonstrated positive clinical significance in anaesthesia for painless gastroscopy. The combination of remimazolam and esketamine not only meets the requirements for appropriate sedation and analgesia but also provides stable haemodynamic parameters. However, esketamine is metabolised more slowly, thus increasing patient recovery time. Wei et al. found that esketamine combined with remimazolam achieved good sedation and stable haemodynamics, but recovery time was prolonged, which is consistent with the results of this study^36^.

There are some limitations to this study. The primary objective was to determine the ED50 of remimazolam for painless gastroscopy at different esketamine doses. Therefore, the sample size was generated based on the sequential method, which may have been insufficient for comparisons of secondary outcome indicators. Future studies with larger sample sizes are warranted to validate the secondary outcomes. In additions, our study observed that a small dose (0.2 mg/kg) of esketamine significantly increased the time to awakening in patients, and future studies should further explore the effects of lower doses (< 0.2 mg/kg) of esketamine on time to awakening and hemodynamics. In addition, while our study focused on hemodynamic stability and common perioperative adverse events, we did not systematically monitor psychiatric adverse effects of esketamine (e.g., hallucinations, nightmares, or dissociative reactions). This oversight limits the comprehensive safety evaluation of the esketamine-remimazolam combination. Future studies should incorporate validated tools to quantify psychotomimetic effects and correlate them with esketamine doses.

## Conclusion

The ED50 of remimazolam combined with 0 mg/kg, 0.2 mg/kg, and 0.4 mg/kg of esketamine for induction of anaesthesia during painless gastroscopy was 0.344, 0.289, and 0.193 mg/kg, respectively. Esketamine combined with remimazolam for induction of anaesthesia during painless gastroscopy was advantageous with respect to haemodynamics and adverse effects.

## Data Availability

The datasets generated and analyzed during the current study are available from the corresponding author upon reasonable request.
